# Supplementary material for: Lack of APOL1 in proximal tubules of normal human kidneys and proteinuric APOL1 transgenic mouse kidneys
Source: PLoS One. 2021 Jun 17;16(6):e0253197. doi: 10.1371/journal.pone.0253197 (PMC8211208; doi:10.1371/journal.pone.0253197)
Supplement: S1 Data — (PDF) [file pone.0253197.s001.pdf]

Supplemental Figures and Tables

APOL1 is not expressed in proximal tubules and is not filtered.

Supplemental Figure 1. Commercially-available monoclonal anti-APOL1 antibodies non-specifically react with epitopes in both normal (non-transgenic wild-type) and APOL1 transgenic mouse kidney sections.

Supplemental Figure 2. APOL1 expression in liver of humans and BAC-APOL1 transgenic mouse models.

Supplemental Table 1A. List of commercial antibodies examined.

Supplemental Table 1B. Summary of commercial anti-human APOL1 antibody testing in Western blotting.

Supplemental Table 1C. Summary of commercial anti-human APOL1 antibody testing in human kidney tissue, and in mouse kidney tissue as a negative control.

Supplemental Detailed Methods.

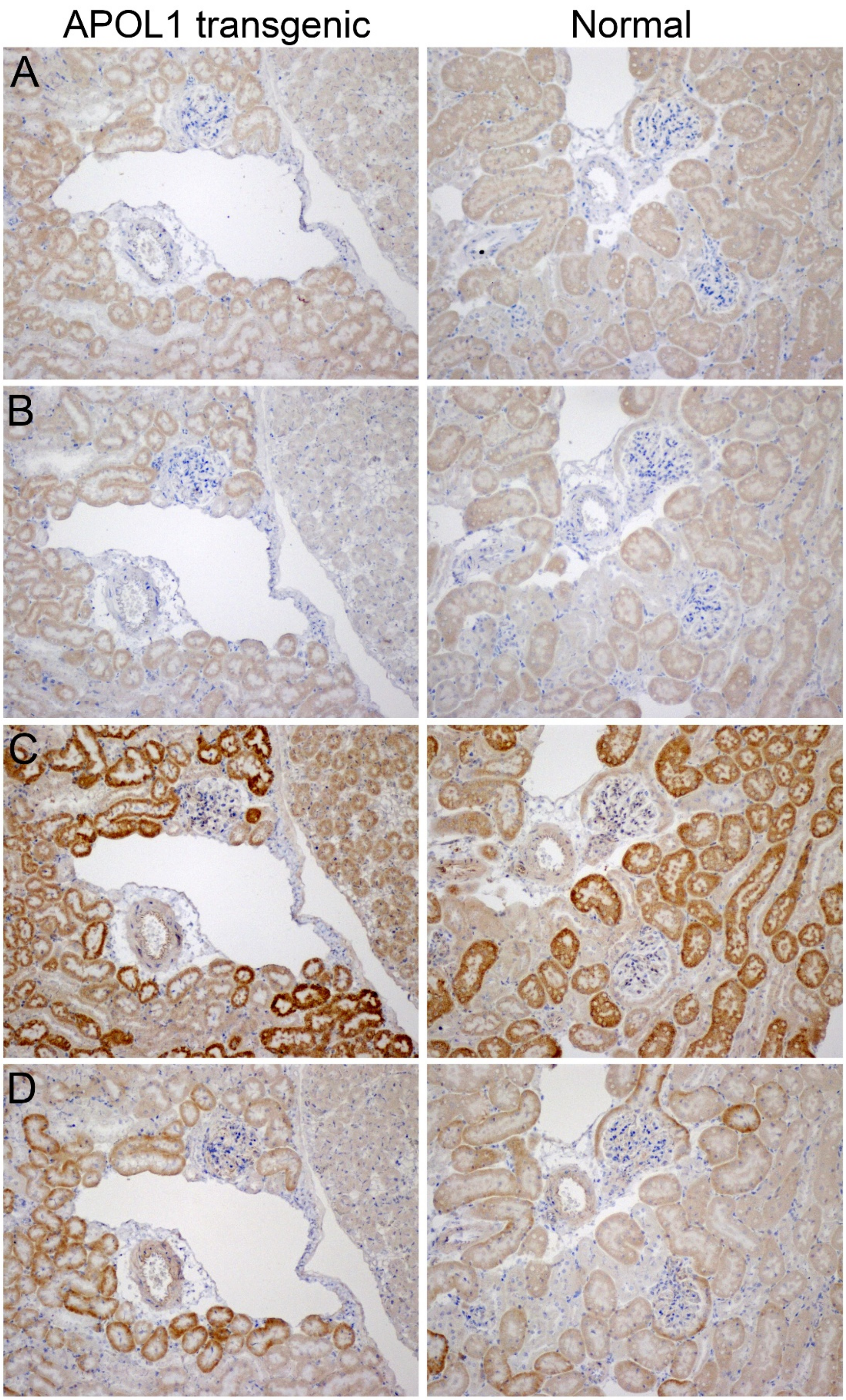

Supplemental Figure 1.

Supplemental Figure 1. Commercially-available monoclonal anti-APOL1 antibodies non-specifically react with epitopes in both normal (non-transgenic wild-type) and APOL1 transgenic mouse kidney sections. Normal mouse kidney does not express APOL1, thus any positive signal seen in normal mouse kidney is artifact. Serial sections for normal mouse and APOL1 transgenic mouse kidneys are shown with hematoxylin counterstain. Conditions for the above images were overnight formalin fixation with boiling citrate buffer antigen retrieval, using an alkaline phosphatase-conjugated secondary antibody and an avidin-biotin amplification step, generating a brown color reaction product (see Supplemental Detailed Methods). **A**. ProteinTech mouse monoclonal (66124-1-Ig). **B**. Sigma mouse monoclonal (AMAB90530). **C**. Epitomic rabbit monoclonal (n-terminal, 2840-1) **D**. Epitomics rabbit monoclonal (c-terminal, 3245-1). See **Supplemental Tables 1A-C** for a summary of other conditions tested and antibody validation results.

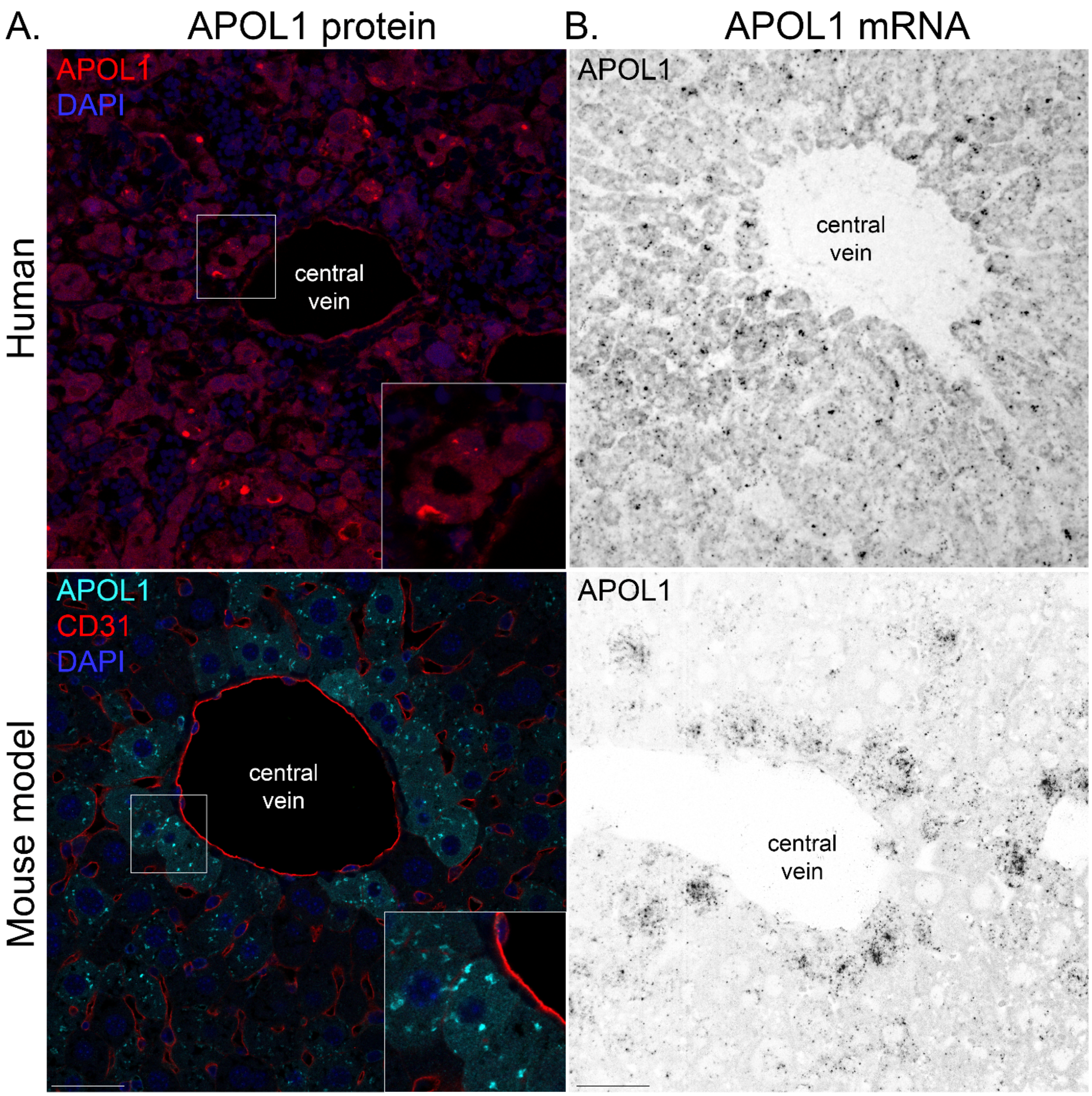

Supplemental Figure 2. APOL1 expression in liver of humans and in the BAC-APOL1 transgenic mouse models. A. Immunostaining for APOL1 protein in human liver (top panels) and BAC-APOL1 transgenic mouse model liver (bottom panels). Mouse section is shown with co-immunostaining for CD31 to indicate endothelial cells, and DAPI as a nuclear counterstain. Expression was evident in hepatocytes, most predominately in zone 3 immediately adjacent to central veins ("CV"), and in the vascular endothelia. **Inset**: APOL1 was present diffusely in the cytoplasm with more intense staining accumulation in intracellular vesicles which were identified as Golgi by co-immunostaining with the Golgi marker GM130 (not shown). **B**. *APOL1* mRNA expression using the RNAscope *in situ* hybridization method confirming expression patterns were consistent with protein expression patterns. The *in situ* hybridization signal appears as dots. Scale bar=40μm.

Supplemental Table 1A. Commercial anti-human APOL1 antibodies tested in our lab.

Antibody # for Table 1B,C	Company (Catalog #)	Type	Immunogen
	Sigma (HPA018885)	**rabbit polyclonal**	aa261-385 (c-terminal)
1A	lot A39110 *		
1B	lot C57797 *		
1C	lot E96151 *		
1D	lot E114503 *		
1E	lot E105900 *		
1F	lot G115599 *		
1G	lot E105260 **		
2	Sigma (AMAB90530)<br>also sold by other vendors	**mouse monoclonal**<br>clone CL0170	aa261-385 (c-terminal)<br>(mapped to aa368-382)
3	Epitomics (3245-1)/Abcam (ab108315)	**rabbit monoclonal**<br>clone EPR2907	aa305-330 (c-terminal)
4	Epitomics (2840-1)/Millipore (MABS387)<br>also sold by other vendors	**rabbit monoclonal**<br>clone EPR2906 or ID4	aa65-90 (n-terminal)
5	ProteinTech (66124-1-lg)	**mouse monoclonal**<br>clone 1G12D11	near full length (238aa)<br>n-terminal GST fusion

* lot has been depleted and is no longer available from manufacturer

** lot used for figures in this manuscript

Supplemental Table 1B. Summary of commercial anti-human APOL1 antibody testing in Western blotting.

anti-APOL1 Antibodies (see Table 1A for code)	Western blotting: dilutions used	detects APOL1 (40-42kDa doublet)	detects other bands
1A	1:500-8000	yes	45kDa[1,5], 50-60kDa[1,2,4], 110kDa[2,4,5]
1B	1:500-8000	yes	45kDa[1], 50-60kDa[1,5], 110kDa[5]
1C	1:500-8000	yes	
1D	1:500-8000	yes	50-60kDa[2,3]
1E	1:500-8000	yes	50-60kDa[4]
1F	1:500-8000	yes	50-60kDa[4]
1G	1:500-8000	yes	50-60kDa[3]
2	1:1000-2000	yes	
3	1:1000-2000	yes	35kDa[1], 50-60kDa[1,2,4,5], 110kDa[1,2,5]
4	1:1000-2000	yes	50-60kDa[4], 80kDa[4], 25kDa[3]
5	1:1000-2000	yes	25kDa[3,4,5]

[1]human proximal tubule cell lines

[2]human serum

[3]CHO cells

[4]human podocyte cell lines

[5]mouse whole kidney/glomerular extract

Supplemental Table 1C. Summary of commercial anti-human APOL1 antibody testing in human kidney tissue, and in mouse kidney tissue as a negative control.

		Immunohistochemistry and/or Immunofluorescence							
		Reactivity in human kidney				Reactivity in normal mouse kidney			
anti-APOL1 Antibodies (see Table 1A for code)	dilutions used	POD	ENDO	PEC	PT	POD	ENDO	PEC	PT
1A	1:200-400	++	++	-	+++	-	-	-	-
1B	1:200-400	+	-	-	++	-	-	+	+
1C	1:200-400	++	-	-	-	-	-	+	-
1D	1:200-400	++	-	-	-	-	-	-	+
1E	1:200-400	++	-	-	-	-	-	-	+
1F	1:200-400	++	-	-	-	-	-	-	+
1G	1:200-400	++	+	+	-	-	-	+	-
2	1:50-100	-	-	-	+	-	-	-	+
3	1:50-100	+++	+	-	+	-	+	-	++
4	1:50-100	-	+	-	++	-	-	-	+++
5	1:50-100	++	++	-	+++	-	-	+	+

POD: podocytes; negative: -

ENDO: endothelium, vasculature; weak: +

PEC: parietal epithelial cells; moderate: ++

PT: proximal tubule; strong: +++

SUPPLEMENTAL DETAILED METHODS:

Western blot protocol

Tissue or cell lysates (25 μg) in Laemmli buffer were resolved by denaturing polyacrylamide gel electrophoresis using 4-20% gradient Tris-glycine gels followed by electrophoretic transfer to polyvinylidene difluoride membranes. Membranes were blocked with 5% nonfat dry milk in PBS with 0.2% Tween-20 at room temperature for 1 hr and incubated with anti-APOL1 antibody (1:500 to 1:8000 dilution in 5% nonfat dry milk in PBS with 0.2% Tween-20) overnight at 4°C. Membranes were washed with shaking three times in PBS containing 0.2% Tween-20, 10 min each wash, probed with secondary antibody (Horseradish peroxidase-conjugated goat anti-rabbit/mouse IgG) at 1:20,000 dilution for 1hr at room temperature, and washed three additional times with shaking in PBS containing 0.2% Tween-20. Immunoreactive proteins were identified using Supersignal West Pico Chemiluminescent Substrate per manufacturer's protocol (ThermoFisher Scientific).

Immunohistochemistry/Immunofluorescence protocol

Fixation conditions tested: Neutral-buffered formalin (10%, Sigma-Aldrich), HistoChoice MB (EMS), Zinc Formalin (Z-fix, EMS), Acetic methanol (50% methanol, 10% acetic acid in water).
Antigen retrieval methods tested: No antigen retrieval, EDTA Antigen Retrieval Buffer (pH=9.0, Abcam), Citrate Buffer Antigen Retrieval (pH=6.0, EMS), Trilogy (Sigma-Aldrich).

Fresh kidney tissue was placed in one of the fixatives listed above, at 4°C overnight. Following fixation, tissues were dehydrated and blocked in paraffin using standard procedures. Paraffin blocks were cut into 4 μm sections and deparaffinized using Clear-Rite (Richard-Allen Scientific), except for Trilogy which is a combined deparaffinization and antigen retrieval process. Deparaffinized sections were processed with one of the antigen retrieval methods above, all using the standard protocols provided by the manufacturer.

Following antigen retrieval, sections processed for immunohistochemistry used an avidin-biotin amplification process with horseradish peroxidase secondary antibodies (ABC kit, Vector Labs). Kit recommended conditions were followed for all steps including blocking endogenous peroxidases. Detection was with DAB chromogen and sections were counterstained with hematoxylin. For immunofluorescence, sections were blocked using 5% normal goat serum in PBS with 0.02% Tween-20 using sufficient volume to cover the tissue section and incubated at room temperature for 1 hr. Without letting the slides dry, slides were tipped to drain off blocking solution and primary antibodies were added, diluted in PBS with 1% normal goat serum (see Supplemental Table 1C for dilutions). Primary antibodies were incubated overnight at 4°C in a humidified chamber. Slides were submerged in wash buffer and washed three times (2 washes in PBS with 0.2% Tween-20, 1 wash in PBS), for 10 min each wash. Secondary antibodies were diluted 1:400 (goat anti-rabbit or goat anti-mouse with appropriate fluorophore, Vector Labs) in 1% normal goat serum in PBS, and incubation was for 1 hr at room temperature in a humidified chamber. Slides were submerged in wash buffer and washed three times (2 washes in PBS with 0.2% Tween-20, 1 wash in PBS) for 10 min each wash. Slides were dipped 10 times in ultrapure water to removed salts and mounted in Vectashield anti-fade mounting media with DAPI (Vector Labs). Alternatively, dehydrating the slides and use of a hard-set anti-fade mounting medium was also successful.
