# Supplementary material for: Lack of APOL1 in proximal tubules of normal human kidneys and proteinuric APOL1 transgenic mouse kidneys
Source: PLoS One. 2021 Jun 17;16(6):e0253197. doi: 10.1371/journal.pone.0253197 (PMC8211208; doi:10.1371/journal.pone.0253197)
Supplement: S1 Raw images — (PDF) [file pone.0253197.s002.pdf]

Figure 5A Coomassie (mouse urine)

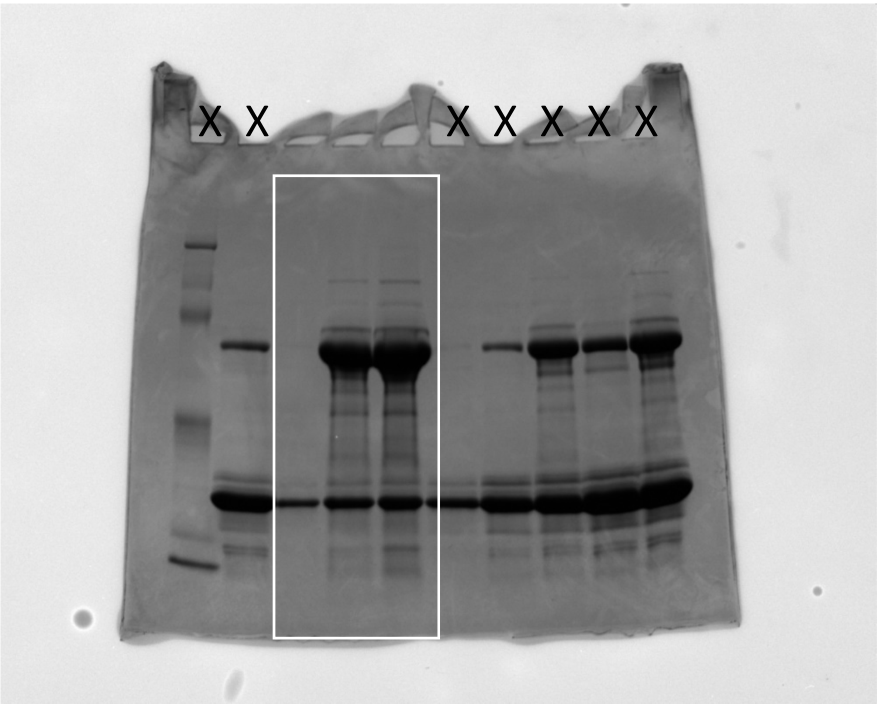

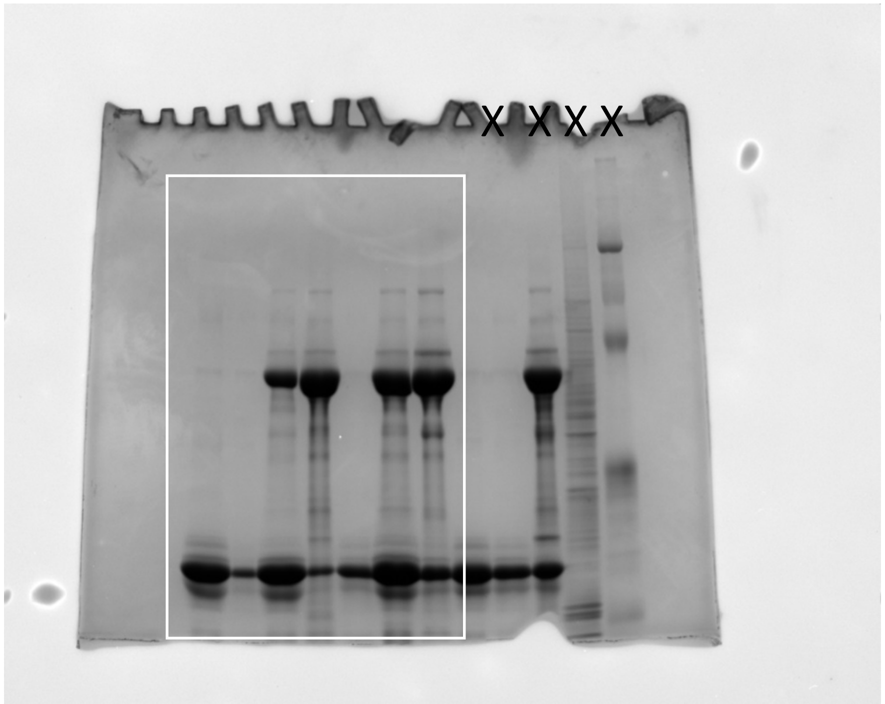

Images acquired using iBright CL1000 imager
(cropping in white box)

Figure 5B APOL1 Western (mouse serum)

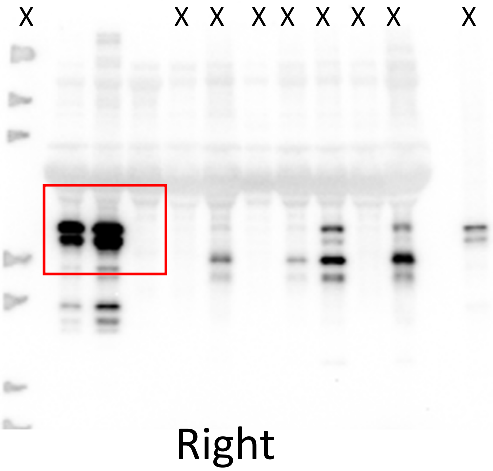

Right

Image acquired using iBright CL1000 imager (cropping in red box)

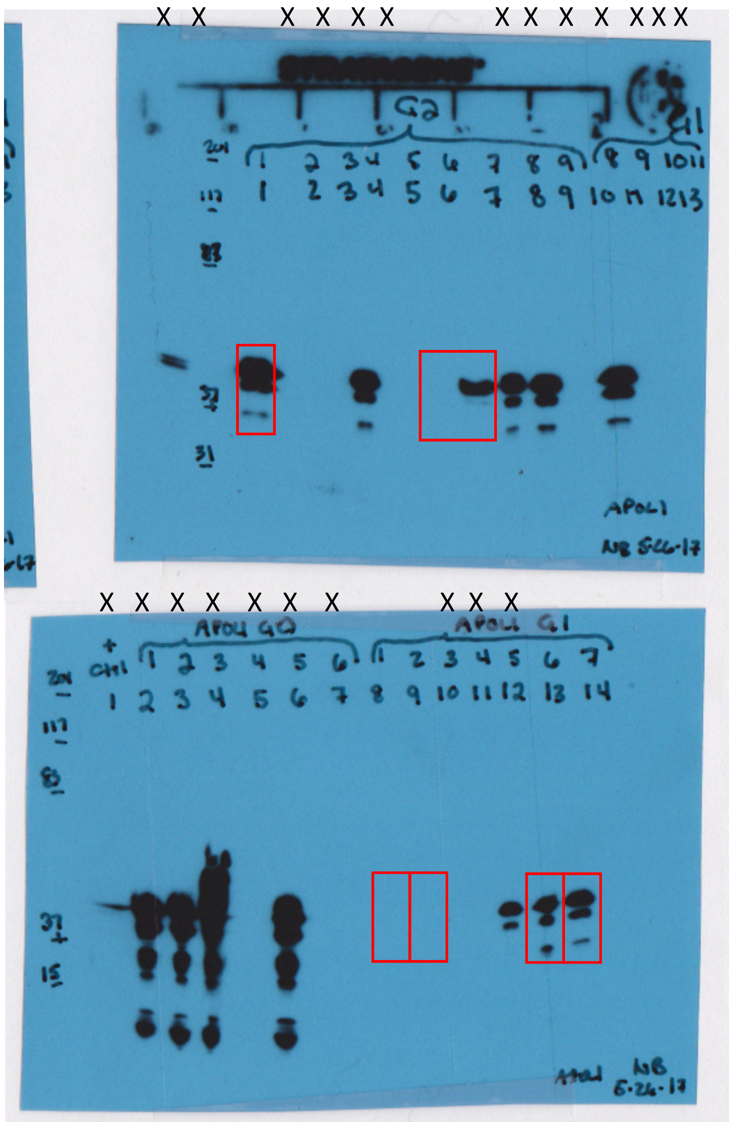

Image acquired by scanning X-ray film with a flat bed scanner (cropping in red box)

Figure 5B APOL1 and APOA1 Westerns (mouse serum)

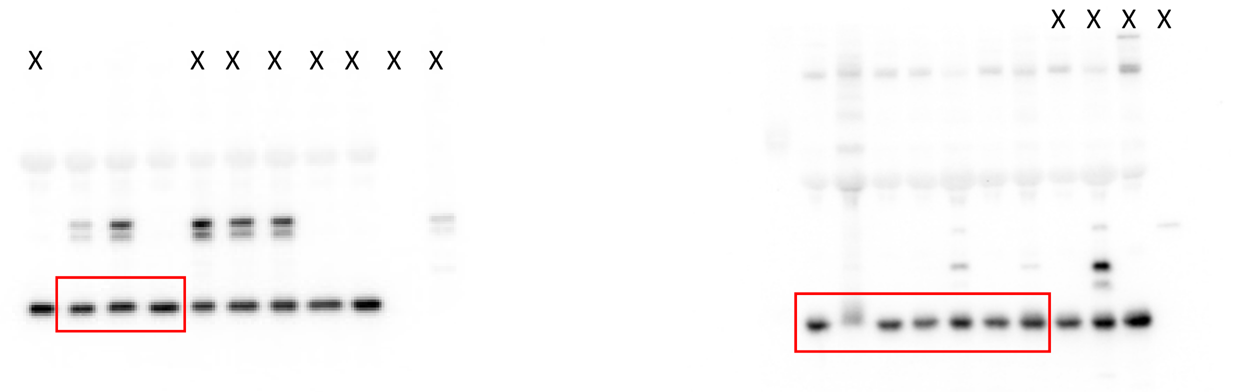

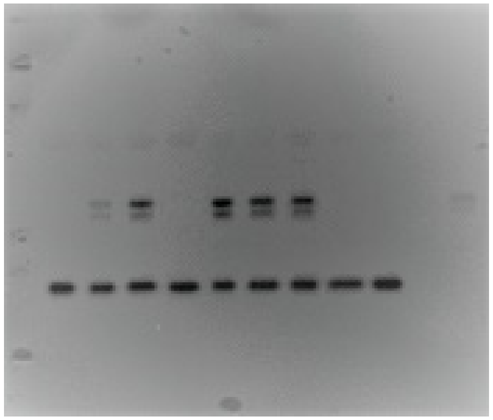

Same gels as above but showing blot overlay for molecular weight markers

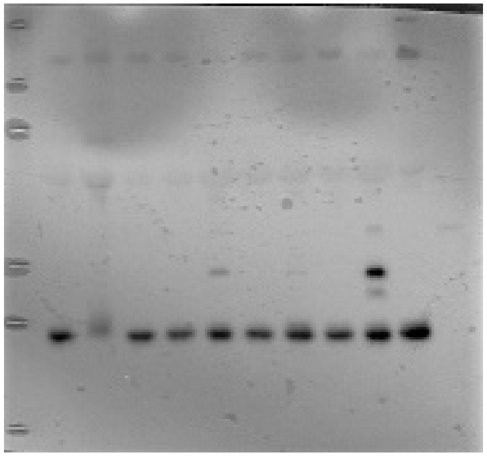

Images acquired using iBright CL1000 imager (cropping in red box)
